# Perioperative sodium bicarbonate to prevent acute kidney injury after cardiac surgery: a multicenter double-blind randomized controlled trial

**DOI:** 10.1186/cc12351

**Published:** 2013-03-19

**Authors:** A Haase-Fielitz, M Haase, M Plass, P Murray, M Bailey, R Bellomo, S Bagshaw

**Affiliations:** 1 Otto von-Guericke University, Magdeburg, Germany; 2 German Heart Center, Berlin, Germany; 3 University College, Dublin, Ireland; 4 ANZIC, Melbourne, Australia; 5 Austin Hospital, Melbourne, Australia; 6 University of Alberta, Canada

## Introduction

Evidence suggests a nephroprotective effect of urinary alkalinization in patients at risk of acute kidney injury (AKI).

## Methods

In a multicenter, double-blind, RCT we enrolled 350 adult cardiac surgery patients. At induction of anesthesia, patients received either 24 hours of intravenous infusion of sodium bicarbonate (5.1 mmol/kg) or sodium chloride (5.1 mmol/kg). The primary endpoint was the proportion of patients developing AKI.

## Results

**Figure 1:**
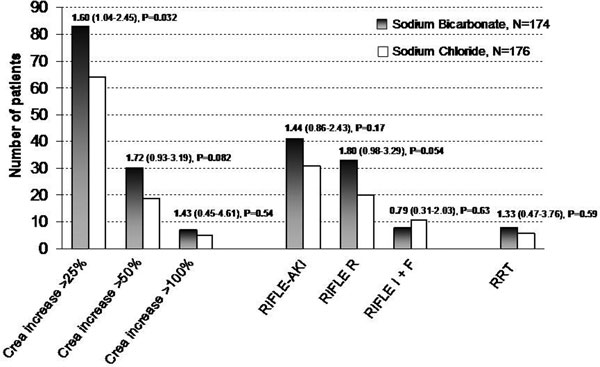


Sodium bicarbonate increased urinary pH (from 6.0 to 7.5, *P *0.001). More patients in the bicarbonate group (83/174 (47.7%)) developed AKI compared with control (64/176 (36.4%), OR = 1.60 (95% CI, 1.04 to 2.45); unadjusted *P *= 0.032). A greater postoperative increase in urinary NGAL in patients receiving bicarbonate infusion was observed compared with control (*P *= 0.011). The incidence of postoperative RRT was similar but hospital mortality was increased in patients treated with bicarbonate compared with chloride (11/174 (6.3%) vs. 3/176 (1.7%), OR 3.89 (1.07 to 14.2), *P *= 0.031). See Figure 1.

## Conclusion

On this basis of our findings we do not recommend the use of perioperative infusions of sodium bicarbonate to reduce the incidence or severity of AKI in this patient group.

